# Incidental Appendiceal Basidiobolomycosis in a Clinical Setting of Intestinal Intussusception in a Five-Year-Old Patient: A Case Report

**DOI:** 10.7759/cureus.31392

**Published:** 2022-11-11

**Authors:** Mohammed O Barasheed, Reem Althubaiti, Bayan Hafiz, Elaf Damanhouri, Fadwa Altaf

**Affiliations:** 1 Pathology, King Abdulaziz University Faculty of Medicine, Jeddah, SAU

**Keywords:** pediatric surgical pathology, pediatric gi surgery, pediatric intussusception, appendiceal intussusception, appendiceal inflammation, gastrointestinal basidiobolomycosis

## Abstract

Gastrointestinal basidiobolomycosis (GIB) is a rare, critical fungal infection caused by *Basidiobolus ranarum*, an environmental saprophyte with a wide geographical distribution. It usually affects the immunocompetent host and presents with nonspecific clinical signs and symptoms, posing a diagnostic and therapeutic challenge. The coexistence of GIB and intussusception is rare, and it is far more unusual for appendiceal basidiobolomycosis and intussusception to coexist.

Herein, we report a case of a five-year-old male who presented to the emergency department with a clinical and radiological picture of intestinal intussusception, for which he underwent laparoscopic exploration and reduction. The appendix was observed to be partially invaginated through the cecum and was difficult to be reduced. Subsequent histopathological examination of the laparoscopically resected appendix demonstrated fungal organisms morphologically consistent with basidiobolomycosis. The patient achieved full recovery with a combination of surgery and prolonged antifungal therapy.

## Introduction

Basidiobolomycosis is a rare fungal infection caused by *Basidiobolus ranarum* (*B. ranarum*), which belongs to the Entomophthorales order of the Basidiobolomycetes class [1,2], and was initially described in 1956 as a case involving a subcutaneous lesion in a four-year-old male in Indonesia [3]. The condition has been reported to affect both children and adults, most commonly affecting immunocompetent patients [4,5]. The majority of basidiobolomycosis cases have been diagnosed in tropical and subtropical regions with warm, humid climates; many animals in such areas are assumed to be carriers of such fungi [2]. *Basidiobolus ranarum* is an environmental saprophyte found in decaying vegetable matter, soil, and the alimentary tract of infected amphibians (e.g., frogs), reptiles (e.g., lizards and geckos), horses, dogs, and bats [4,6]. Basidiobolomycosis primarily presents as a subcutaneous tissue infection involving the extremities, trunk, or buttocks. The disease is thought to be acquired after a minor skin injury or an insect bite. Consuming food contaminated by infected soil or animal feces is thought to result in the less common gastrointestinal basidiobolomycosis (GIB) [7].

GIB was first described in a case involving infection of the stomach and transverse colon of a four-year-old male in Brazil in 1980 [8]. Gastrointestinal tract involvement is rare; however, the trend in the literature suggests increasing GIB incidence. A recent review of worldwide cases indicated a high infection rate among patients living in Saudi Arabia, as well as in Iran and the United States, with most cases involving male children [9].

The clinical presentation of GIB can be nonspecific; the most common presentations include abdominal pain, followed by fever, weight loss, abdominal mass, vomiting, and diarrhea [10]. Accordingly, the differential diagnosis includes various neoplastic (i.e., lymphoma or carcinoma) and nonneoplastic (inflammatory bowel disease and infectious diseases such as amebiasis, intestinal tuberculosis, or sarcoidosis) conditions [11]. Misdiagnosis of GIB is common. Most reported cases were initially evaluated as entities mimicking common visceral diseases such as Crohn’s disease and colon cancer [12]. Reports have frequently indicated a strong association between infection, peripheral blood eosinophilia, and high erythrocyte sedimentation rate (ESR) [11,13].

Herein, we report a unique case of pediatric GIB involving the appendix coexisting with intestinal intussusception.

## Case presentation

A five-year-old male who resides in the southern region of Saudi Arabia with a history of bronchial asthma and eczema presented with a four-month history of recurrent abdominal pain and fever but without vomiting or diarrhea; he received oral antibiotics, but his condition failed to improve. Subsequently, he presented to the King Abdulaziz University Hospital’s emergency department with substantially worsened abdominal pain associated with frequent bowel motion, decreased oral intake, reduced activity, and foul-smelling breath. A history of animal contact, including with sheep and cats, was also obtained.

On examination, the patient was conscious, oriented, alert, and not in distress. The patient was placed comfortably on the bed, and vital signs were monitored. His vital signs were as follows: temperature, 36.8°C; pulse, 114 beats per minute; blood pressure, 110/76 mmHg; respiratory rate, 22 per minute; and oxygen saturation (SpO2), 100%. His abdomen was soft and lax, with no tenderness on palpation or palpable mass. Chest examination revealed clear bilateral equal air entry. The cardiovascular examination revealed normal heart sounds. Initial laboratory tests yielded the following results: white blood cell (WBC) count, 11.51 K/μL, with peripheral eosinophilia (11.9%); hemoglobin, 11.6 g/dL; platelet count, 532 K/μL; and C-reactive protein, 34 mg/L. Other test results were unremarkable, including those for blood electrolytes, urine analysis, urine culture, and blood culture. Abdominal ultrasonography revealed ileocolic and colocolic intussusception up to the proximal and mid-transverse colon associated with bowel wall thickening, ileocecal mass, and small bowel obstruction.

Based on the clinical and radiological findings, a diagnosis of intussusception was made, and the patient underwent emergent surgery. Upon laparoscopic exploration, the intussusception was identified as ileocolic, and reduction was attempted until the cecum was identified; the appendix was observed to be partially invaginated through the cecum and was difficult to be reduced. A laparoscopic appendectomy was performed, and the appendix was sent for histopathological examination.

**Figure 1:**
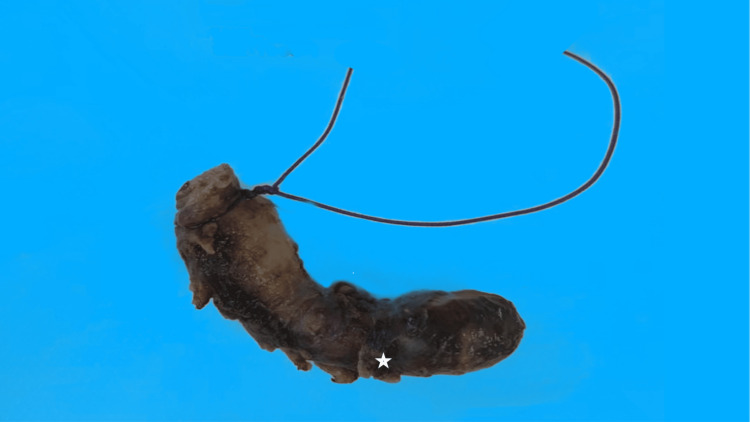
Inflamed appendix exhibiting dark red-brown congested outer surface (asterisk).

**Figure 2:**
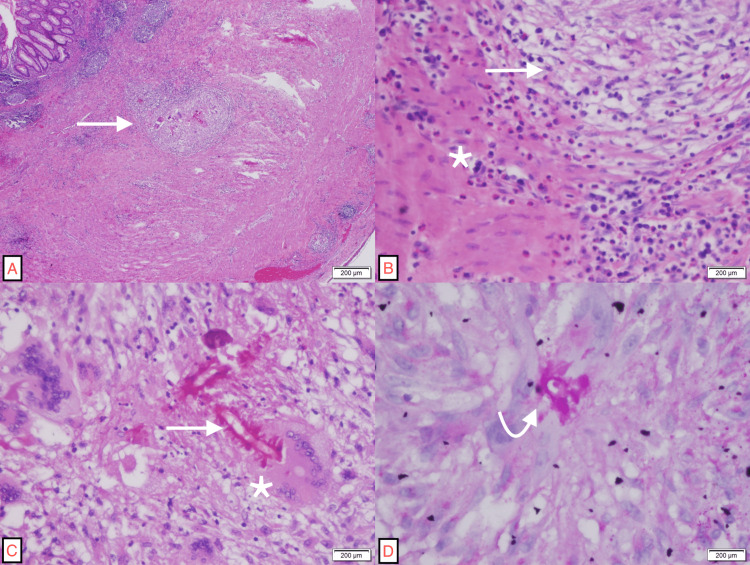
(A) Low-power view of the appendiceal wall exhibiting granulomatous inflammation (arrow) (H&E, 20×). (B) Appendiceal muscularis propria showing prominent eosinophilic infiltrate (asterisk) and granulomatous reaction (arrow) (H&E, 400×). (C) High-power view of a granuloma exhibiting fungal organisms featuring broad non-septate hyphae surrounded by dense eosinophilic reaction, the Splendore-Hoeppli phenomenon (arrow), partially engulfed by a multinucleated giant cell (asterisk) within the granuloma (H&E, 400×). (D) Transverse section of a fungal hypha (curved arrow) with the characteristic Splendore-Hoeppli phenomenon (PAS, 400×). H&E: hematoxylin and eosin, PAS: periodic acid-Schiff

The specimen, consisting of a congested appendix measuring 6.0 × 1.0 cm, was fixed in 10% neutral buffered formalin, and upon examination, it exhibited a dark-brown outer surface (Figure 1). Hemorrhage and blood clots were observed in the appendiceal lumen. Sections were taken for histopathological examination. Microscopically, the appendiceal wall was heavily infiltrated by eosinophils, with multiple granulomas evident throughout the appendiceal wall layers (Figure 2A, 2B). In some granulomas, fungal broad, non-septate hyphae surrounded by a sunburst pattern of dense eosinophilic reaction, the Splendore-Hoeppli phenomenon, were observed. Multinucleated giant cells partially engulfing fungal organisms were seen (Figure 2C). Special studies, including Grocott’s methenamine silver (GMS) stain and periodic acid-Schiff (PAS) stain, highlighted the microorganisms (Figure 2D). Ziehl-Neelsen stain for acid-fast bacilli was negative.

Based on the microscopic findings, the patient was diagnosed with appendiceal basidiobolomycosis. Accordingly, the patient was initially started on a daily 50 mg amphotericin B intravenous infusion for one day. After that, he was shifted to 128 mg voriconazole pediatric infusion twice daily for three weeks and then to 150 mg oral voriconazole twice daily for up to 12 months with regular radiological and laboratory test follow-up. A postoperative computed tomography (CT) scan showed persistent ileocecal intussusception with thickened bowel and ileocecal valve with submucosal edema, forming a mass. The imaging study was discussed with the pediatric surgery team, as this bowel thickening could represent a fungal ball.

Throughout the patient’s stay in the hospital for one month, he was subjected to various laboratory tests to ensure his ability to tolerate treatment, and CT scan and ultrasonography studies to ensure postoperative improvement. The patient’s vital signs, liver function tests, complete blood count (CBC), and blood electrolytes were regularly monitored. Two weeks after the operation, the liver function test started to reveal an increase in liver enzymes as follows: alanine aminotransferase (ALT) from 14 to 219 U/L, aspartate aminotransferase (AST) from 20 to 146 U/L, and gamma-glutamyl transferase (GGT) from 12 to 98 U/L. The patient at that time showed jaundice, which disappeared with time as the liver enzyme levels started to decline within days. Before the patient’s discharge from the hospital, he was vitally stable with an unremarkable physical examination. He did a series of CT scans and ultrasounds, which eventually revealed no signs of recurrence of intussusception or bowel obstruction.

Upon the recent follow-up visit to the clinic, the patient was active and doing fine, with no signs of distress. The abdominal examination was unremarkable, with no signs of tenderness on palpation. Laboratory tests, including CBC, urea and electrolytes, ESR, and liver function test, were ordered and showed the following results: WBC, 7.90 K/μL; hemoglobin, 11.9 g/dL, peripheral eosinophils, 6.5%; normal electrolytes; normal ESR; elevated alkaline phosphatase (ALP), 256 U/L; normal ALT; normal AST; and normal GGT. A follow-up abdominal ultrasound and CT scan with contrast were ordered, and the reports showed a normal appearance of the terminal ileum and ileocecal valve, with no evidence of residual bowel wall thickening or intussusceptions. The patient was supplied with oral voriconazole home medication, and the next follow-up visit was booked for him.

## Discussion

To the best of our knowledge, this is only the second reported case of GIB coexisting with intestinal intussusception in Saudi Arabia. Overall, only two prior cases have shown an association between GIB and intussusception [13]. The first reported case of intussusception secondary to GIB was in a 23-month-old immunocompetent male from Iran [11]. A second recently published report highlighted a similar association in a four-year-old male from Saudi Arabia; in that case, the diagnosis was confirmed through a colonic biopsy [13].

Basidiobolomycosis often presents as cutaneous infections that manifest as subcutaneous nodules or hyperpigmentation primarily affecting the back, buttocks, and lower extremities; however, visceral tissue involvement, most commonly in the gastrointestinal tract, is an emerging phenomenon [14,15]. A review study of 122 cases found that the infection rate of basidiobolomycosis was highest in Saudi Arabia (50.8%), followed by Iran (19.8%), the United States (17.2%), and Iraq (4.9%), with most patients being male children [9]. Several authors have noted that most GIB cases have been diagnosed, as in our case, in the southern region of Saudi Arabia, which has a warm and humid climate that can increase fungal growth with consequent environmental contamination [16].

The clinical presentation of GIB is typically challenging to diagnose and not specific. In a systematic review of 18 pediatric cases of GIB in Saudi Arabia, Shreef et al. found that abdominal pain (94.4%), constipation (83.3%), abdominal mass (77.8%), and fever (22.2%) were the most common clinical presentations. In most of the reviewed cases, GIB was mistaken for Crohn’s disease or cancer, as GIB may form a mass that can spread to another organ [17]. In a cohort study by Vikram et al., the presence of a colon or rectal mass in imaging studies was the most frequent finding [18]. Similarly, in 12 cases diagnosed between 2012 and 2019, all patients presented with a palpable abdominal mass on physical examination [15]. Due to the lack of a characteristic clinical or radiological presentation and the overlap of clinical signs and symptoms with various other conditions, the main challenge in diagnosing GIB is its consideration during the initial evaluation of presenting patients [13]. In most reported cases, the patients have undergone surgery for a mistaken clinical diagnosis or an exploratory laparotomy before a subsequent biopsy, or the pathological examination prompted the diagnosis of GIB [10]. Our case presents a similar diagnostic challenge in which the patient had a four-month history of episodic abdominal pain and fever, which was ineffectively managed with oral antibiotics. As a result of the lack of an accurate, timely diagnosis, the patient’s condition worsened, and intestinal intussusception developed. Hence, it is essential to develop a strong clinical suspicion of this emerging fungal infection, especially in patients living in endemic regions with a history of animal contact and peripheral eosinophilia, and consider this disease in the differential diagnosis during the workup of such high-risk patients with intestinal intussusception.

Histopathological examination can establish the diagnosis of GIB; however, the gold standard of diagnosis is to culture *B. ranarum* from clinical or surgical specimens or isolate the organism through molecular detection using polymerase chain reaction (PCR) [2,11]. The most common microscopical findings are non-septate fungal hyphae with the characteristic, albeit nonspecific, Splendore-Hoeppli phenomenon, giant cell granulomatous inflammation, and eosinophilic cell infiltrate, as in our case [2,13]. Furthermore, the infection usually affects the non-mucosal layers of the gut; superficial endoscopic biopsy may reveal nonspecific inflammation, making the disease diagnosis even more challenging [19].

The mainstay of treatment is prolonged antifungal medication, with surgery as a vital adjunct therapy [5,13]. The common antifungal agents administered are itraconazole, voriconazole, and posaconazole [13,18]. The use of amphotericin B has been associated with therapeutic failure and resistance in some cases [13]. Our patient has been on oral voriconazole for eight months with good tolerance. In a clinical study on 58 pediatric patients aged nine months to 15 years with invasive fungal infections such as aspergillosis, candidiasis, and scedosporiosis treated with voriconazole, Walsh et al. reported a high success rate (62%) among patients with chronic granulomatous diseases, as in our case. Treatment-related side effects were observed in 23 of the selected patients. The most commonly observed treatment-related side effects were elevated liver transaminases or bilirubin (n = 8), skin rash (n = 8), abnormal vision (n = 3), and a photosensitivity reaction (n = 3) [20]. As in our patient’s case, the treatment’s early side effect was an elevation in the liver transaminases, which was associated with jaundice. Thus, regular liver function tests are performed to ensure no voriconazole-related liver function abnormalities.

## Conclusions

GIB is an often-misdiagnosed fungal illness due to its rarity and nonspecific clinical presentation. Further studies are needed to address the predisposing factors of this infection in immunocompetent patients, as well as the treatment options and their side effects. This case report can alert clinicians to this emerging fungal infection, raising awareness of its unusual presentation and encouraging early recognition and diagnosis, especially in endemic regions. Clinicians should be suspicious of GIB in cases presenting with vague abdominal pain and abdominal masses associated with unexplained peripheral eosinophilia. Prompt detection and proper clinical and surgical management are essential for ensuring positive outcomes for patients.
